# Delineating Human B Cell Precursor Development With Genetically Identified PID Cases as a Model

**DOI:** 10.3389/fimmu.2019.02680

**Published:** 2019-11-26

**Authors:** Marjolein W. J. Wentink, Tomas Kalina, Martin Perez-Andres, Lucia del Pino Molina, Hanna IJspeert, François G. Kavelaars, Arjan C. Lankester, Quentin Lecrevisse, Jacques J. M. van Dongen, Alberto Orfao, Mirjam van der Burg

**Affiliations:** ^1^Department of Immunology, Erasmus MC, University Medical Center Rotterdam, Rotterdam, Netherlands; ^2^Department of Paediatric Haematology and Oncology, Second Faculty of Medicine, Charles University and University Hospital Motol, Prague, Czechia; ^3^Department of Medicine-Service Cytometry, Cancer Research Center (IBMCC-CSIC/USAL) and University of Salamanca, Salamanca, Spain; ^4^Department of Clinical Immunology, La Paz University Hospital, Lymphocyte Pathophysiology in Immunodeficiencies Group La Paz Institute for Health Research (IdiPAZ), Madrid, Spain; ^5^Department of Hematology, Erasmus MC, University Medical Center Rotterdam, Rotterdam, Netherlands; ^6^Department of Immunohematology and Blood Transfusion, Leiden University Medical Center, Leiden, Netherlands; ^7^Department of Pediatrics, Leiden University Medical Center, Leiden, Netherlands

**Keywords:** next generation sequence (NGS), immunoglobulin repertoire, bone marrow, flow cytometry, precursor B-cell

## Abstract

B-cell precursors (BCP) arise from hematopoietic stem cells in bone marrow (BM). Identification and characterization of the different BCP subsets has contributed to the understanding of normal B-cell development. BCP first rearrange their immunoglobulin (Ig) heavy chain (IGH) genes to form the pre-B-cell receptor (pre-BCR) complex together with surrogate light chains. Appropriate signaling via this pre-BCR complex is followed by rearrangement of the Ig light chain genes, resulting in the formation, and selection of functional BCR molecules. Consecutive production, expression, and functional selection of the pre-BCR and BCR complexes guide the BCP differentiation process that coincides with corresponding immunophenotypic changes. We studied BCP differentiation in human BM samples from healthy controls and patients with a known genetic defect in V(D)J recombination or pre-BCR signaling to unravel normal immunophenotypic changes and to determine the effect of differentiation blocks caused by the specific genetic defects. Accordingly, we designed a 10-color antibody panel to study human BCP development in BM by flow cytometry, which allows identification of classical preB-I, preB-II, and mature B-cells as defined via BCR-related markers with further characterization by additional markers. We observed heterogeneous phenotypes associated with more than one B-cell maturation pathway, particularly for the preB-I and preB-II stages in which V(D)J recombination takes place, with asynchronous marker expression patterns. Next Generation Sequencing of complete IGH gene rearrangements in sorted BCP subsets unraveled their rearrangement status, indicating that BCP differentiation does not follow a single linear pathway. In conclusion, B-cell development in human BM is not a linear process, but a rather complex network of parallel pathways dictated by V(D)J-recombination-driven checkpoints and pre-BCR/BCR mediated-signaling occurring during B-cell production and selection. It can also be described as asynchronous, because precursor B-cells do not differentiate as full population between the different stages, but rather transit as a continuum, which seems influenced (in part) by V-D-J recombination-driven checkpoints.

## Introduction

B cells arise from hematopoietic stem cells in bone marrow (BM) and develop in a stepwise manner (1, 2). Identification and characterization of the different B-cell precursor (BCP) subsets contributes to the understanding of normal B-cell development (3, 4). Currently, it is accepted that expression of the PAX5 transcription factor triggers commitment to the B-cell lineage through the expression of B-cell specific genes such as CD79a (or Igα) and CD19, while suppressing B-lineage inappropriate genes. Since cytoplasmic expression of CD79a (cyCD79a) is one of the first signs of B-cell lineage commitment, human cyCD79a+ CD19- cells are defined as pro-B cells, followed by CD19 expression in pre-B-I cells. During this stage, V-D-J recombination of the immunoglobulin (Ig) heavy chain (IGH) locus is initiated by the recombination activating genes (RAG1 and RAG2) (5–11). If this rearrangement process results in a functional protein, Igμ heavy chain is expressed in the cytoplasm (cyIgμ), which defines the pre-BII-stage. Whenever rearrangement of the first allele does not result in a productive Igμ molecule, the second allele will be rearranged. Igμ is expressed on the cell membrane together with the surrogate light chains λ14.1 and VpreB as pre-B-cell receptor (pre-BCR) (12, 13). Pre-BCR signaling triggers a cascade of events including downregulation of the recombination machinery to ensure allelic exclusion and subsequent proliferation, followed by opening of the Ig light chain (IGL) locus, which is being rearranged under the influence of a second expression wave of RAG1 and RAG2 (10, 14). After successful IGL rearrangement, a functional BCR in the form of a complete IgM molecule is expressed on the cell surface membrane, defining progression to the immature B-lymphocyte stage. Subsequent IgD expression on the plasma membrane of IgM^+^ immature B-cell leads to the differentiation into mature naive B-lymphocytes, which are released from BM to peripheral blood (PB). In addition to the above described changes in rearrangement status and expression of Ig molecules, BCP undergo also other maturation-associated immunophenotypic changes. Whereas, Pro-B cells express stem cell markers such as CD34 and CD10, later stages start to express B-cell specific markers such as CD19 and CD20. Additionally, cells that are in their rearrangement process express TdT in two waves, one during the IGH gene rearrangements and another during the IGL gene rearrangement which ensures junctional diversity by random addition of non-templated nucleotides at the joining sites of the V, D, and J genes. Expression of these markers and the different variants of the immunoglobulin (BCR) complex-molecules can be studied with flow-cytometry (15).

Most knowledge about B cell development in BM came from mouse studies; however, detailed insight into normal human BCP development is important to identify and unravel pathophysiological processes in hematological malignancies and primary immunodeficiencies (PID), caused by genetic defects (16). In turn, BCP analysis in genetically-defined PID can help elucidating the role of specific genes in BCP development (13, 17), because absence (or dysfunction) of essential proteins cause a full or incomplete block of maturation at specific developmental stages (18–20).

Here, we studied BCP differentiation in BM from both healthy controls and patients with a well-defined genetic defect in V(D)J recombination or pre-BCR signaling, to further unravel the normal immunophenotypic profiles of BM BCP at distinct stages of maturation and to determine the type of differentiation blockades caused by specific genetic defects. In multiple cycles of design, testing, evaluation, and redesign, we developed a 10-color antibody combination and applied novel data analysis strategies based on multivariate (principal component and viSNE) analysis (21) that allowed more detailed characterization of previously described BCP populations. This 10-color antibody combination was first validated against a conventional 4-color panel (7, 15, 18, 22). Secondly, we analyzed Ig gene rearrangement status and the gating strategy based on BCR-associated markers (cyCD79a, cyIgμ, IgM, IgD, CD19) was compared with gating based on membrane markers such as CD10 and CD20, as also done in the literature (7, 23–25). Gating based on BCR-associated markers allowed us to define the crucial steps of B cell development better than gating based on other non-BCR-associated surface markers alone, while intracellular markers emerged as essential to adequately delineate BCP development.

## Materials and Methods

### Bone Marrow Samples

BM samples from healthy controls were left over samples from healthy children who donated BM for transplantation into a diseased sibling or were collected from patients that had a BM biopsy to rule out other diseases than lymphoid PID. The latter BM samples were considered to be normal when no malignant cells were detected in combination with a normal BCP differentiation pattern upon standard diagnostic testing. Patient BM samples were collected for PID-diagnostics. Both normal BM samples and patient BM samples in this study were obtained with informed consent according to the guidelines of the local medical ethics committee of the Erasmus MC (MEC-2013-026) and the LUMC (P08.001).

### Flow Cytometric Immunophenotyping and Repertoire Analysis of Bone Marrow

Flow cytometric immunophenotyping of BM samples was performed on a LSR Fortessa (BD Biosciences, San Jose, CA) with instrument setting according to EuroFlow SOP (26). Following the EuroFlow bulk-lysis SOP (26, 27), cells were stained for surface membrane (sm) and intracellular markers in two consecutive steps. The following surface stainings -fluorochrome conjugate (clone)- were used: IgM-BV510 (MHM-88) CD38-BV605 (HIT2) and CD20-PB (2H7) were all from Biolegend (San Diego, CA); CD34-APC (8G12) and IgD-PeCF594 (IA6) were both from BD Biosciences); CD19-PC7 (J3-119) was purchased from Beckman Coulter (Fullerton, CA); and CD10-APC-C750 (HI10a) was obtained from Cytognos (Salamanca, Spain). Sustained cells were fixed and permeabilized using the Fix&Perm reagent kit (An der Grub, Vienna, Austria) according to manufacturer's instructions, and further stained for intracellular markers: IgM-PerCPcy5.5 (MHM-88) from Biolegend; TdT-FITC (HT6) purchased from (Supertechs, Rockville, MD); and CD79a-PE (HM47) purchased from Beckman Coulter) (see Table 1 for complete panel).

**Table 1:** Composition and technical information on reagents of the 10-color EuroFlow BCP tube.

**Fluorochrome**	**PB**	**BV510**	**BV605**	**FITC**	**PE**	**PE-CF594**	**PerCP-Cy5,5**	**PE-Cy7**	**APC**	**Alexa750**
Target	CD20	IgM	CD38	TdT	CD79a	IgD	cyIgM	CD19	CD34	CD10
clone	2H7	MHM-88	HIT2	HT6	HM47	IA6	MHM-88	J3-119	8G12	HI10a
Volsume (undiluted)	1 μl	1.3 μl	1 μl	10 μl	5 μl	3 μl	2.5 μl	5 μl	2.5 μl	5 μl

*Shaded fields indicate intracellular markers*.

Patient BM samples were analyzed in parallel with a diagnostic 4-color panel as previously described (7, 18), for comparison of the new 10-color panel to the 4-color diagnostic panel (gold standard). Based on the 4-color protocol the main precursor B-cell populations were defined as follows: pro-B-cells as CD22^+^CD19^−^; pre-B-I cells as CD19^+^cyIgμ^−^, pre-B-II cells as CD19^+^cyIgμ^+^IgM^−^; immature cells as CD19^+^IgM^+^IgD^−^ and CD19^+^IgM^+^IgD^+^. For calculation the composition of the precursor B-cell compartment mature B-cells are excluded, because mature B-cells can also arise from peripheral contamination. An overview of the antibodies used in all panels can be found in Table 1.

For data was analysis with Infinicyt (Version 1.8, Cytognos) and Cytobank (Cytobank, Inc, Santa Clara, CA, USA) software programs were used. Principle component analysis was performed with the Infinicyt software. This method calculates the most discriminating projections based on selected parameters, into a single Automated Population Separator (APS) bi-dimensional graph. Multiple APS graphs (APS1, APS2 etc.) can be generated, depending on which parameters contribute more or less to the principle components on the X-axis and Y-axis. ViSNE projection (21) was calculated using Cytobank (Cytobank, Inc, Santa Clara, CA, USA) (28). This method generates a 2D dotplot in which the X- and Y-axis are defined by virtual parameters called tSNE1 and tSNE2, in which all events are projected integrating information on all selected parameters. In a viSNE plot the distance of one event to other events represents how similar events are, with the most similar events plotting closest to each other (21).

For repertoire analysis, two BM samples without malignant cells and with normal BCP differentiation (determined with standard diagnostic testing) were enriched for B cells using a RosetteSepp human B-cell enrichment cocktail (Stem cell Technologies, Vancouver, Canada) according to the manufacturer's instructions, as described elsewhere (29). Subsequently, the B-cell enriched samples were frozen in liquid nitrogen and thawed prior to sorting. Sorting was done with the same 10-color antibody combinations as described above on an FACS Aria-III flow cytometer (BD Biosciences). After sorting, cells were washed and DNA was isolated using a direct lysis procedure as described elsewhere (30). From this DNA, *IGH* rearrangements were amplified in a 2-step PCR and sequenced by NGS. *IGH* rearrangements were amplified (35 cycles) using the forward VH1-6 FR2 and reverse JH consensus EuroClonality/BIOMED-2 primers, extended with Illumina P5 and P7 adapter sequence (31). Subsequently, PCR products were purified by gel extraction (Qiagen, Valencia, CA), followed by a nested PCR reaction (12 cycles) to include the sample-specific indices and Illumina sequencing adapters using primers from the Illumina TruSeq Custom Amplicon Index Kit (Illumina, San Diego, CA). The final PCR product concentration was measured using the Quant-it Picogreen dsDNA assay (Invitrogen, Carlsbad, CA). The libraries were analyzed by NGS (221 bp paired-end) on the MiSeq platform (Illumina, San Diego, CA, USA) with use of an Illumina MiSeq Reagent Kit V3, according to the manufacturer's protocol (Illumina, San Diego, CA, USA). Paired sequences were aligned using paired-end read merger (PEAR) (32), and the fastq files were converted to fasta files (33). Subsequently, the sequences were trimmed to remove the primer sequence and uploaded in IMGT/High-V-Quest (34); subsequently, the IMGT output files were analyzed using the ARGalaxy tool (https://bioinf-galaxian.erasmusmc.nl/argalaxy) (35). For analysis only a single sequence per clone (defined as same V gene, same J gene and the nucleotide sequence of the CDR3 region) were included. In-frame IGH rearrangements were defined to have an in-frame rearrangement without a stop codon. Unproductive IGH rearrangements were either out-of-frame rearrangements or in-frame rearrangements with a stop codon.

## Results

### Subset Definition Based on BCR-Associated Markers Is Consistent Between Different Panels

**Figure 1:**
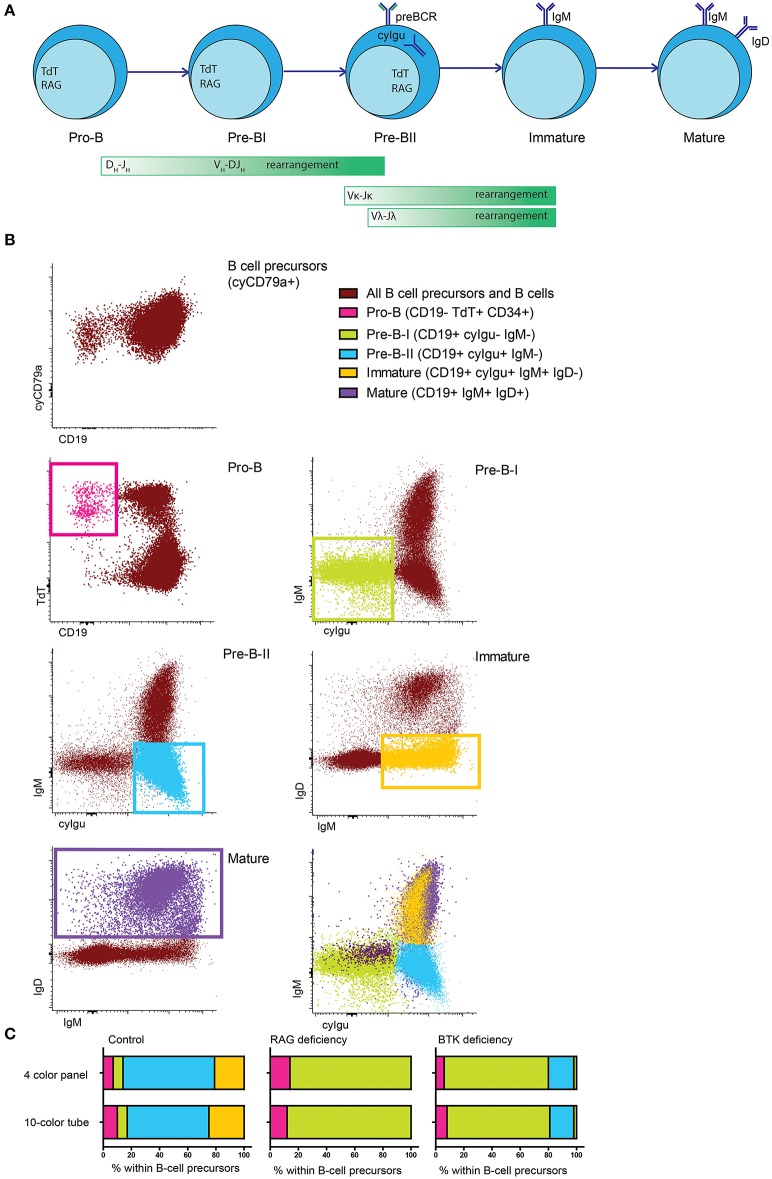
Major BCP subsets in human bone marrow. **(A)** Schematic representation of the BCP subsets in human bone marrow, the green bars indicate when recombination processes take place. **(B)** Population definition based on BCR-related markers. All cyCD79a expressing cells are considered BCP or B cells. Pro-B cells are defined as CD19- TdT+, pre-BI cells are defined as CD19+ cyIgμ- IgM-, pre-BII cells are defined as CD19+ cyIgμ+ IgM-, immature B cells are defined as CD19+ IgM+ IgD- and mature B cells are defined as CD19+ IgM+ IgD+. **(C)** BCP subset distribution in the same sample that was acquired in parallel with two different panels. Population definition was in both cases done as indicated above.

To study human BM, we designed and validated a 10-color flowcytometry antibody combination to be stained in a single tube (Table 1), to make optimal use of available material and integrate information about both intracellular and extracellular markers on each individual cell. This 10-color tube was tested against a previously validated 4-color diagnostic panel (7, 18) using BM samples from healthy controls and PID patients. B cells and BCP were defined as cyCD79a+. The five major B-cell populations (pro-B, pre-BI, preB-II, immature and mature B cells) (Figure 1A) were gated based on the staining profiles for the BCR-associated markers CD19, nTdT, cyIgμ, IgM, and IgD (Figure 1B and Supplementary Material), as defined by the previously observed subset distribution with the 4-color panel used as gold standard. Since IgMD^+^ cells (mature B cells) can also be detected in peripheral blood (PB), they were not considered as a formal BCP stage. In ten independent (*n* = 4 controls and 6 patients) samples both panels revealed the same precursor B-cell subset distribution, as illustrated by three representative cases in Figure 1C: one of normal BCP development, a RAG deficient patient and a BTK deficient patient. This indicates that gating based on BCR-associated markers is consistent between both panels and gives comparable results in both healthy controls and PID patients with defects in BCR signaling or V(D)J recombination (Figure 1C).

### B-Cell Populations Defined Based on BCR-Associated Markers Only Show Heterogeneous Intra-Population Phenotypes

**Figure 2:**
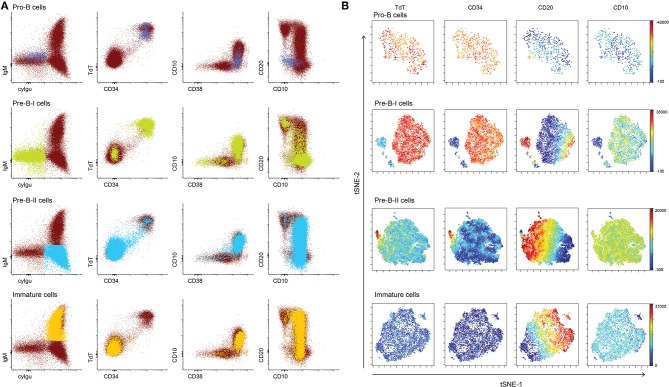
Heterogeneity within BCP subsets. **(A)** Dotplots of a representative normal bone marrow sample, showing all cyCD79a+ B cells in bone marrow. Populations of interest are highlighted in a different color: pro-B cells in pink, pre-B-I cells in green, pre-B-II cells in blue, immature cells in orange. **(B)** Results of a viSNE analysis of individual B-cell precursor subsets, represented by tSNE-1 vs. tSNE-2 plots. The color scale represents the intensity of the stain for individual markers: TdT, CD34, CD20, CD10, and CD38.

Based on BCR-associated markers only (cyCD79a, CD19, cyIgμ, IgM, and IgD), 4 distinct subsets of BCP were identified/defined as described above. Further analysis of the expression profiles for other markers (i.e. TdT, CD34, CD10, CD20, and CD38) within these four B-cell populations showed highly heterogeneous patterns, particularly within pre-BI and pre-BII BCP (Figure 2A). Thus, pre-BI BCP were mainly TdT+ and CD34+, but some cells had lost one or both markers, while, at the same time, they were CD10+ and CD20- ruling out they could be unswitched memory B-cells. Similarly heterogeneous patterns of expression were observed for CD10 and CD38 (most cells being positive but a minority negative) and CD20 (most cells CD20- but some were CD20+), pointing out the existence of multiple subsets of pre-BI cells (Figure 2A). In turn, pre-BII cells, were cyIgμ-positive while mostly negative for CD34 and TdT, but with some CD34+ and TdT+ pre-B-II cells. CD20 expression was highly heterogeneous within this population with progressively more CD20 molecules per cell (Figure 2A). Multivariate (e.g., viSNE) analysis confirmed the presence of minor subsets of TdT− CD34− pre-BI B-cells and CD34+ TdT+ pre-BII cells, both populations showing progressively higher expression levels of CD20, similarly to immature B-lymphocytes (Figure 2B).

### Asynchronous Expression of Non-BCR-Associated Markers in BCP Stages Defined by BCR-Associated Patterns

**Figure 3:**
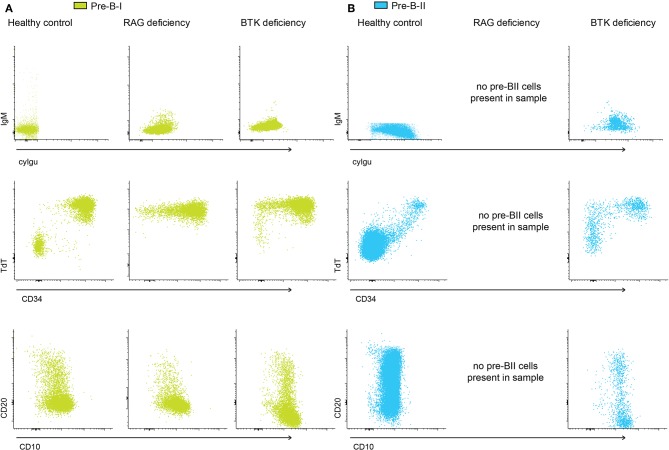
pre-BI and pre-BII marker expression in control and patient samples. **(A)** Dot plots representing pre-BI cells from a control sample, and two patient samples, indicating the uncoupling of expression of cyIgμ and surface markers like CD34, CD20, and CD10. In both the control and the patients, cells without cyIgμ can lose CD34 expression and gain CD20 expression. **(B)** Dot plots representing pre-BII cells from a control sample, and two patient samples. In both the control and the patients, cells with cyIgμ expression can still express CD34 and TdT, and CD20 expression is heterogeneous.

In order to gain further insight into the relationship between the pattern of expression of non-Ig related markers and cyIgμ, we compared BM pre-BI and pre-BII cells from controls to patients with RAG-deficiency and BTK-deficiency. As described above a fraction of all pre-BI cells in controls, loses CD34 and/or TdT expression, and some upregulate CD20 (Figure 3). In RAG-deficient patient, TdT expression remains intact, but loss of CD34 together with some upregulation of CD20 was observed. Thus, it appears that BCP can lose CD34 and upregulate CD20 in the absence of a functionally rearranged heavy chain while they do not loose TdT. In BTK-deficiency a similar profile was observed. In addition, within the pre-BII BCP of controls and BTK-deficient patients, some cells retained CD34 and/or TdT expression, although they already expressed cyIgμ. Since RAG deficient patients do not have pre-BII cells, we could not compare this subset for these patients. Altogether, there results point out the existence of different kinetics of expression of BCR-associated and other non-BCR-related markers during normal B-cell maturation. Additionally, RAG or BTK deficient cells can lose CD34 expression, without downregulating TdT.

### Dissection of Multiple BCP Maturation Pathways in BM

**Figure 4:**
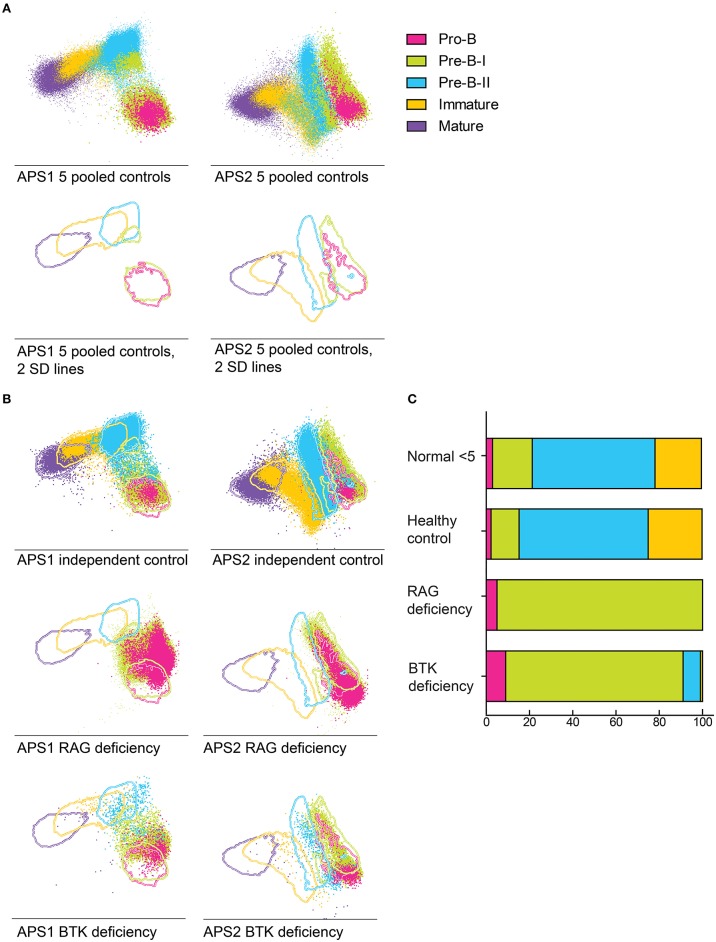
Supervised APS view of BCP populations in healthy bone marrow (*n* = 5) indicated by lines at 2SD intervals. **(A)** BCP populations in a pool of 5 healthy bone marrow samples that were used to create the APS view. Lines indicate the 2 SD range of each population, dot indicate individual cells. The different populations are indicated by the different colors. **(B)** 2 SD lines of BCP populations derived from a pool of 5 healthy donors, dots indicate total BCP from a sixth healthy donor, a RAG deficient patient and a BTK deficient patient, plotted against the reference pool. **(C)** Bars indicate the BCP subset distribution of each sample, compared to age matched controls (<5 years).

Multivariate analysis of normal BM BCP based on all markers evaluated simultaneously revealed the existence of up to three (parallel) distinct maturation pathways, where the BCR-associated cyIgM and CD20 represent the major discriminating markers (Supplemental Figure 1). Based on a data set of 5 healthy control BM samples, a reference BM profile was built using the APS1 view –Principal Component (PC) 1 *vs*. PC2- of the in Infinicyt software (36) (Figure 4A), after plotting the 2SD lines corresponding to each reference population of normal BCP. Once this reference profile has been built, BCP events from a sixth, independent healthy donor was plotted against it (Figure 4B), the events neatly falling within the ranges of the other 5 healthy controls BM samples. In contrast, when BM BCP from a patient with RAG-deficiency and another child with BTK-deficiency were plotted against the reference profile, profiles with a clear blockade appeared with most cells exclusively present within the first maturation pathway. In the BTK deficient patient, a small fraction of the BCP cells reached the pre-B-II stage (Figure 4C). Furthermore, in this representation, some patient cells fell outside the 2SD lines of both pre-BI and pre-BII suggesting the existence of aberrant phenotypic profiles.

### IGH Gene Rearrangement Profile of BCP at the pre-BI and pre-BII Stage Showing Distinct Patterns of Expression of CD34 and TdT

**Figure 5:**
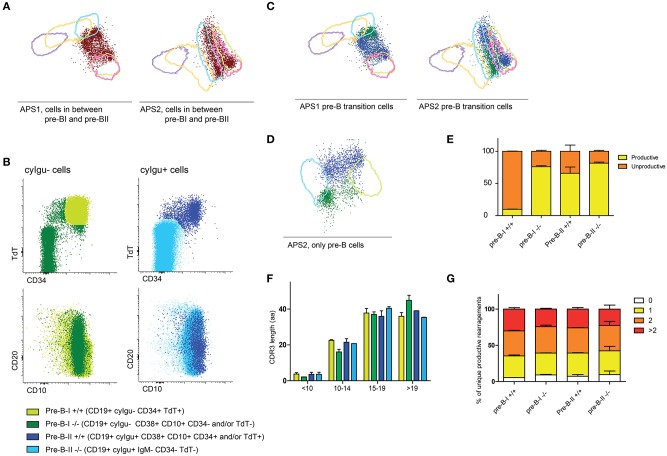
Further dissection of the BCP compartment. **(A)** APS views indicating the cells that are outside the 2SD lines of the pre-B-I and pre-B-II populations of healthy controls. **(B)** Expression of CD34, TdT, CD10, and CD20 within cyIgμ- (in green) and cyIgμ+ cells. All cells here are CD19+ CD10+ CD38+ indicating that they are bone marrow derived B cells. **(C)** APS views indicating the cells that are outside the 2SD lines of the pre-BI and pre-BII populations of healthy controls, color coded as in B. **(D)** APS view supervised on pre-BI and pre-BII cells, separating the transitioning pre-B cells further. **(E)** Distribution of in-frame and out-of-frame DNA-rearrangements in sorted pre-B subpopulations. **(F)** CDR3 length of productive rearrangements of the four sorted pre-B-I and pre-B-II populations. **(G)** The proportion of CDR3 regions with ≥3, 2, 1, or 0 positive charges.

When we focused on the cells that were neither within the 2SD interval of the pre-BI cells nor in the 2SD interval of pre-BII cells, we found that they are present in normal BCP differentiation (between 1 and 3% of all BCP cells), although not as many as in the patient samples (Figure 5A). These cells are defined as pre-BI or pre-BII based on the absence or presence of cyIgμ, but the expression of TdT, CD34, CD10, and CD20 is asynchronous with their cyIgμ status (Figure 5B). We can divide the pre-BI cells in true pre-BI cells that are CD19+ cyIgμ- CD34+ TdT+ (pre-BI+/+) and another, more heterogeneous group that is CD19+ cyIgμ- but where CD34 and or TdT expression is negative (pre-BI–/–). These cells are not switched memory B cells coming from peripheral blood, because they all express both CD10 and CD38. The same split can be made within the pre-BII cells, dividing them in pre-BII cells that are CD19+ cyIgμ+ CD34− TdT− (pre-BII–/–) and a heterogeneous group that is CD19+, cyIgμ+ but that still have CD34, TdT or both these markers (pre-BII+/+). In the APS views that are based on all BCPs, these cells end up between pre-BI and pre-BII. If we create an APS view of only the pre-BI and pre-BII stages, we can examine how heterogeneous these populations are (Figures 5C,D).

### Rearrangement Status Classifies the Intermediate Stages

To further dissect this, we sorted the pre-B populations into four populations: pre-BI +/+ (CD19+ cyIgμ- CD34+ TdT+), pre-BI–/– (CD19+ cyIgμ− CD34- TdT−), pre-BII–/– (CD19+ cyIgμ+ CD34− TdT−) and pre-BII+/+ (CD19+ cyIgμ+ CD34+ TdT+). We isolated DNA from these subsets and sequenced complete IGH rearrangements using next-generation sequencing. We found that in the pre-BI+/+ cells, the majority of complete rearrangements is non-productive with an in-frame: non-productive ratio of 1:9, which is in line with the observation that these cells do not express cyIgu, yet (Figure 5E). In the pre-BII–/– cells, the in-frame: non-productive ratio is 4:1, with ~80% of rearrangements in frame, which is in line with the observation that these cells all express cyIgu. In the pre-BI–/– population, the in-frame: non-productive ratio is 3:1, and in the pre-BII +/+ population, this ratio is also 3:1. In these populations, the relative amount of in-frame complete rearrangements is approximately the same, however, in one population the cells do not express cyIgu, whereas they do in the other populations. This indicates that in the pre-BI–/– cells in-frame rearrangements are present, but they are either not productive (i.e., not leading to a functional protein) or they are not (yet) expressed. The pre-BII +/+ already express cyIgu, but did not yet downregulate CD34 and TdT. In addition, we analyzed the CDR3 lengths of the productive rearrangements as well as proportion of IGH CDR3s with ≥3, 2, 1, or 0 positive charges (Figures 5F,G). The CDR3 lengths of productive rearrangements of pre-B-I–/– cells were longer than the productive rearrangements of pre-B-I +/+ and pre-B-II cells, which could be explained by a lower number of nucleotide deletions. The proportions of IGH CDR3 charges did not differ between the four subpopulations.

## Discussion

In this study, we designed and validated a 10-color flow cytometry panel, to study human BCP development in BM of immune deficient patients at crucial developmental thresholds in more detail than was done previously. In our standardized measurements, we could reliably gate populations according to BCR-related markers. This allowed us to superimpose PID samples over healthy controls and describe the deviations from normal development as found in patients. Unexpectedly, when we included information from additional (non-BCR) markers, we found heterogeneity, especially within the preB-I and preB-II populations, during which V(D)J recombination takes place with expression of surface markers that seem asynchronous to the expression of cyIgμ. NGS analysis of complete IGH rearrangements in sorted populations was used to determine the rearrangement status at the DNA level.

We showed that BCR-related marker based population definition is consistent over samples and different panels, but this results in heterogeneous populations when other markers, like CD34, TdT, and CD20 are considered. Upon more in-depth study of expression patterns of these markers, we found that in some specific populations, expression of these markers is asynchronous to the process of BCR-formation. This effect is more visible in patients with defects in V(D)J-recombination. Specifically, we found that CD20 can be upregulated in the absence of cyIgμ expression and that cells can lose TdT expression and CD34 expression without having expression of heavy chain protein in the cytoplasm.

We combined the population-gating based strategy that is often used in flow cytometry with principle component analysis. We showed that the BCP populations seem to overlap, indicating a continuous process rather that a step-wise differentiation. This is in line with asynchronous marker expression that we see between surface markers e.g. each cell seems to up-and-down regulate its phenotype markers at its own pace. Even more, some phenotype markers that were previously thought to be V(D)J-recombination dependent, seem to progress even in the absence of cyIgμ expression, as indicated by loss of CD34 and gain of CD20 in patients with genetic defects in V(D)J-recombination. Some of the cellular phenotypes that we found in controls are more common in genetically defined patient samples. This indicates that, even though cells cannot successfully rearrange their IgH-locus, as is the case in RAG deficiency (10, 37), they will still lose CD34 expression as if they are progressing to the next stage. In addition to that, we found that CD20 expression is gradually increasing over the course of several stages. However, CD20 expression is heterogeneous in many populations. Especially in patient samples, we often detected high CD20 expression in populations that were assumed to be early in B-cell differentiation. Even though the exact role of CD20 on B cells is not found yet, it is still a useful marker indicating B-cell development and if highly expressed in combination with loss of CD10 and CD38, indicating maturity of the B cells. CD20 expression does not seem to be related with BCR-rearrangement.

Both in PID patients and in control samples, we identified cells outside the reference borders of the defined populations (e.g., pre-BI and pre-BII) in the APS plots, although the cell numbers were much lower in control samples. Especially in the RAG deficient patient this is striking, because it seems as if some cells can progress in surface marker expression by expressing CD20 and loosing CD34, without having a functionally rearranged heavy chain. This further supports the idea that expression of CD20 and CD34 is not in all cells strictly linked to IGH gene rearrangement status.

To further dissect this, we sorted preB-I and preB-II cells and further divided these populations based on their CD34 and TdT expression. DNA extracted from these cell populations was used to study complete IGH-rearrangements using NGS. In preB-I+/+ cells we showed that close to 90% of detectable IGH gene rearrangements are non-productive. Since these cells do not express cyIgu yet, we hypothesize that these cells have not yet obtained an in-frame IGH rearrangement, and thus they still express CD34 and TdT. In preB-II cells that are CD34- TdT-, around 75% of detectable IGH rearrangements were in-frame. These cells all express cyIgu, and some have an non-productive rearrangement on one allele combined with an in-frame rearrangement on the second allele, which explains the 25% of non-productive rearrangements in this population. The preB-II +/+ cells also contain ~75% of in-frame rearrangements. Possibly, these cells have only just completed their in-frame rearrangement, starting already expressing cyIgu but still need to downregulate CD34 and TdT. However, our data is not sufficient to conclude this. Also, we detected around 75% in-frame rearrangements in preB-I–/– cells. These cells have an in frame IGH rearrangement at the DNA level, but they do not (yet?) express cyIgu protein. To further investigate this, single-cell analysis on DNA, RNA and protein level might give further insight in how and why V(D)J recombination status and phenotypic marker expression are linked.

In conclusion, we have designed and validated a standardized 10-color staining and analytical tools for the analysis of BCP compartment in human BM. Our data indicate that BCP differentiation is not a single linear differentiation pathway, but rather a complex process of V(D)J recombination-driven checkpoints, divergence, parallel pathways and convergence to form a unique and functional BCR. The data also support the notion that B cell maturation is asynchronous, implying that precursor B-cells do not differentiate as full population between the different stages, but rather transit as a continuum, which can be influenced in part by V(D)J recombination-driven checkpoints. Due to this continuum, small populations of cells are captured as they transit between stages and, thus, have intermediate patterns of marker expression. Understanding the process of BCP differentiation requires an integrated approach of single-cell DNA, RNA and protein analysis, which can be applied for studying blockades in BCP differentiation pathways of genetically defined immunodeficient patients. We propose that our immunophenotyping panel can be used in multi-center studies with the standardization stringency developed by the EuroFlow consortium (26), thus allowing to mutually compare the data-files generated on patients with primary immunodeficiency with those of individuals with undisturbed B cell development.

## Data Availability Statement

The raw data supporting the conclusions of this manuscript will be made available by the authors, without undue reservation, to any qualified researcher.

## Ethics Statement

The studies involving human participants were reviewed and approved by Normal BM samples and patient BM samples in this study were obtained with informed consent according to the guidelines of the local medical ethics committee of the Erasmus MC (MEC-2013-026) and the LUMC (P08.001). Written informed consent to participate in this study was provided by the participants' legal guardian/next of kin.

## Author Contributions

MB, TK, AO, and JD: contributed conception and design of the study. MW, TK, MP-A, LM, HI, FK, AL, and QL: performed the data acquisition and data analysis. MW and MB wrote the manuscript. All authors contributed to manuscript, read, and approved the submitted version.

### Conflict of Interest

The authors declare that the research was conducted in the absence of any commercial or financial relationships that could be construed as a potential conflict of interest.
