# Supplementary figures and images for: Delineating Human B Cell Precursor Development With Genetically Identified PID Cases as a Model
Source: Front Immunol. 2019 Nov 26;10:2680. doi: 10.3389/fimmu.2019.02680 (PMC6901940; doi:10.3389/fimmu.2019.02680)

Supplemental Material: Extended gating strategy of BMP

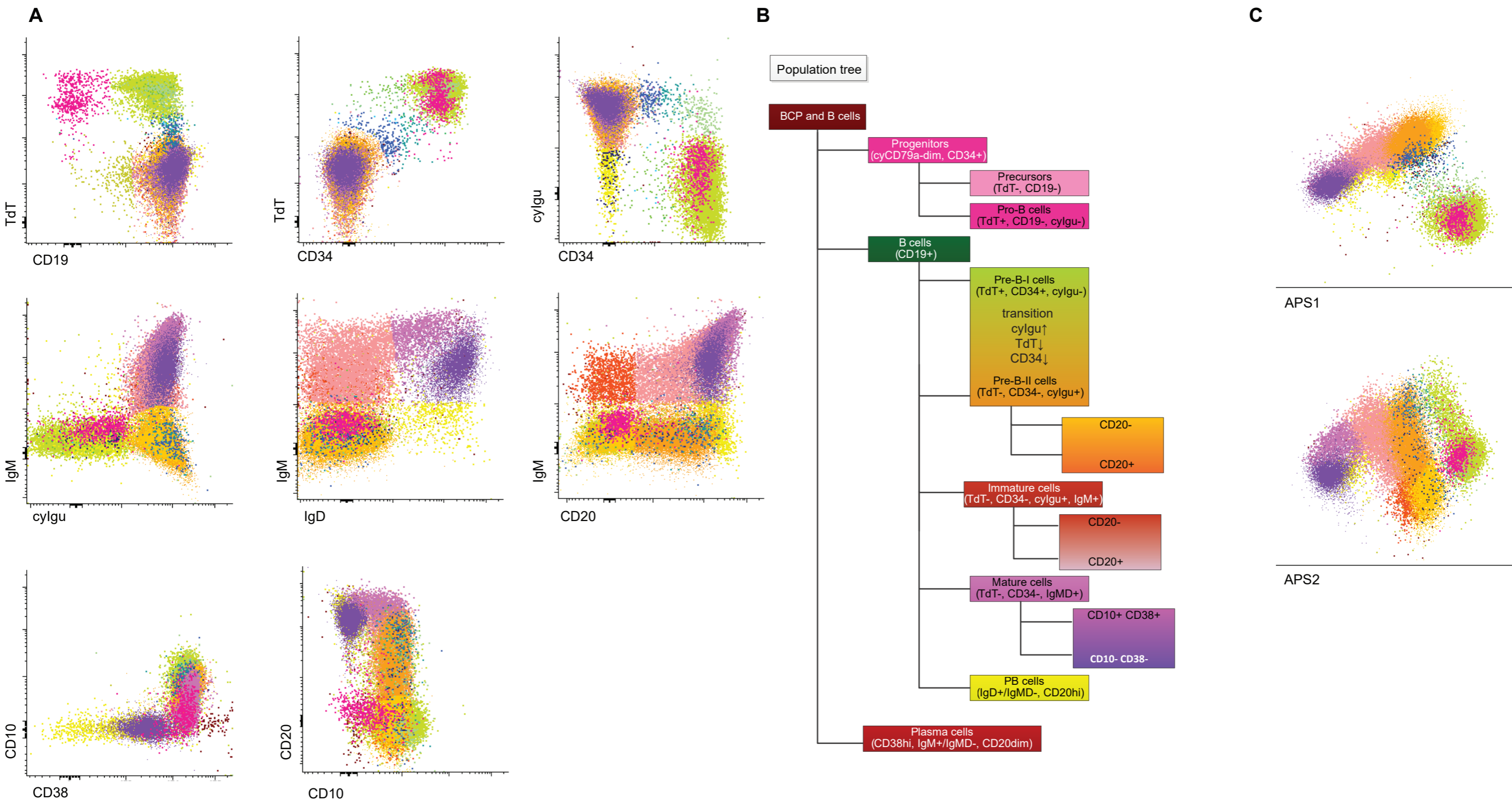

Supplement: Supplementary file 1 [file Image_1.pdf]
